# Effects of Cocrystallization on the Structure and Properties of Melt-Cast Explosive 2,4-Dinitroanisole: A Computational Study

**DOI:** 10.3390/molecules27249010

**Published:** 2022-12-17

**Authors:** Defu Wang, Lei Yang, Weihua Zhu

**Affiliations:** Institute for Computation in Molecular and Materials Science, School of Chemistry and Chemical Engineering, Nanjing University of Science and Technology, Nanjing 210094, China

**Keywords:** DNAN, cocrystallization, melt-cast explosives, intermolecular interactions, melting points

## Abstract

Melt-cast explosive 2,4-dinitroanisole (DNAN) crystal and its cocrystals DNAN/1,3-dinitrobenzene (DNB) and DNAN/2-nitroaniline (NA) were used to identify the effects of cocrystallization on the crystal structure, non-covalent interactions, and melting points of the DNAN crystal through density functional theory and molecular dynamics. The components DNB and NA with subtle structure variations between the nitro group and amino group can significantly affect the non-covalent interactions, especially the π-π stacking and H-bonds, which can lead to different crystal stacking styles. The melting points of the DNAN crystal are decreased through the cocrystallization, which expands the utilization of the DNAN-based melt cast explosives. Our study deciphers the effects caused by the cocrystallization on the structure and properties of melt cast explosives and may help to design and optimize novel melt-cast explosives.

## 1. Introduction

Energetic materials (EMs) are widely used in the fields of military explosives, civilian blasting equipment, or propellants because they can release large amounts of energy during explosion or combustion [1]. However, EMs may explode or decompose unexpectedly under some external stimuli, such as excessive temperature during manufacture, impact during storage and transportation and so on. So, their safety is one of the main topics of concern in the field of EM research [2]. Among the hazardous reduction methods, melt-cast explosives have been noticed. Melt-cast explosives are compounds that have acceptable for manufacturing melting points (*T*_m_, ~100 °C) with no decomposition at this temperature [3]. The explosives with lower *T*_m_ can be easy to transform into a liquid prior to thermal decomposition, which is necessary in the casting of EM. Additionally, melt casting can be used to obtain explosives with high loading densities, particularly when the temperature ranges are large between melting and decomposition [4]. 

Since the first synthesis in 1863, 2,4,6-trinitrotoluene (TNT) has been extensively used as a traditional melt-cast explosive owing to its low *T_m_* (79.05 °C) [5]. Nevertheless, TNT has a series of shortcomings that need improvement, including comparatively low detonation performance, low stability, low mechanical properties, and environmental unfriendliness [3]. Therefore, many efforts have been made to develop the alternatives of TNT. Numbers of mixtures containing TNT with optimized properties have been produced; for instance, Ammonite (80% NH_4_NO_3_, 20% TNT), Octol (75% HMX, 25% TNT) and TNTO (50% TNT, 50% NTO) [3]. New synthetic EMs with higher stability and environmental friendliness are emerging and replacing TNT in the use of melt-cast explosives [3,6], including 2,4-dinitroanisole (DNAN), 1,3,3-trinitroazetidine (TNAZ) [7], etc. 

Cocrystallization has become an increasingly powerful tool for optimizing the properties of explosives since the first energetic cocrystal of benzotrifuroxan/13,14-dithiatricyclo [8,2.1.1^4,7^]- tetradeca-4,6,10,12-tetraene (BTF/DTTD) was synthesized in 1965 [8,9]. It can modulate the properties of EM without destroying the integrity of the original energetic molecules. Both experimental and theoretical research have focused on optimizing the properties of cocrystal explosives. For instance, two DNAN-based cocrystals, DNAN/1,3-dinitrobenzene (DNB) and DNAN/2-nitroaniline (NA), were recently synthesized successfully [10]. The two cocrystals both exhibit better performance (including lower *T*_m_ and higher stability) than their coformers. However, the differences in the properties of the two cocrystals caused by DNB and NA remain unknown. A high-throughput screening method of energetic cocrystals based on thermal analysis was proposed [9]. The report concluded that a lot of novel energetic eutectics (for example, dihydroxylammonium 5,5’-bistertrazole-1,1’-diolate (TKX-50)/18-crown-6) showed relatively low *T*_m_. So, it is necessary to study the modulation rules of the melting point of coformers in order to facilitate the production of more powerful energetic materials. 

It is well known that the density, energy, and structure of an explosive will change abruptly at *T*_m_. Computational prediction of *T*_m_ will benefit the optimization and application of melt-cast explosives. Molecular dynamics (MD) have been used to predict the *T*_m_ of numerous explosives through the turning point in the curve of specific volume vs. temperature. *T*_m_ of trans-1,4,5,8-tetranitro-1,4,5,8-tetraazadacalin (TNAD)-based binary blending explosives were predicted by MD simulations [11]. Solubility differences between different binary blending systems were obtained, and the relationship between intermolecular interactions and solubility was identified. It was found that TNAD/*N*-nitrodihydroxyethylaminedinitrate (DINA) has the highest solubility and intermolecular interactions. Liu et al. modulated the heating velocity of MD to accurately predict the *T*_m_ of nitromethane (NM) [12] and found that the intermolecular interactions affect the distribution of the NM molecules. Although previous studies have predicted the *T*_m_ of DNAN/DNB and DNAN/NA cocrystals, the reasons for the variations between the two cocrystals are unclear. The roles played by the coformers in adjusting the properties of DNAN based melt-cast explosives are a mystery. 

In this work, based on the three crystal structures previously synthesized by Sun et al. [10] and Hiroki et al. [13], we employed density functional theory (DFT) and molecular dynamics (MD) to study the effects of cocrystallization on the stability and melting points of DNAN-based melt-cast cocrystals. Firstly, their crystal structures and crystal packing styles were compared. Then, intermolecular interactions were described in detail to identify the main force for stabilizing the cocrystals. Combined with electronic properties, MD simulations of the melting progress of the two cocrystals were discussed. These insights may provide a guide to the design, optimization, and application of melt-cast explosives.

## 2. Results

### 2.1. Crystal Structure and Stacking

#### 2.1.1. Crystal Structure

**Figure 1:**
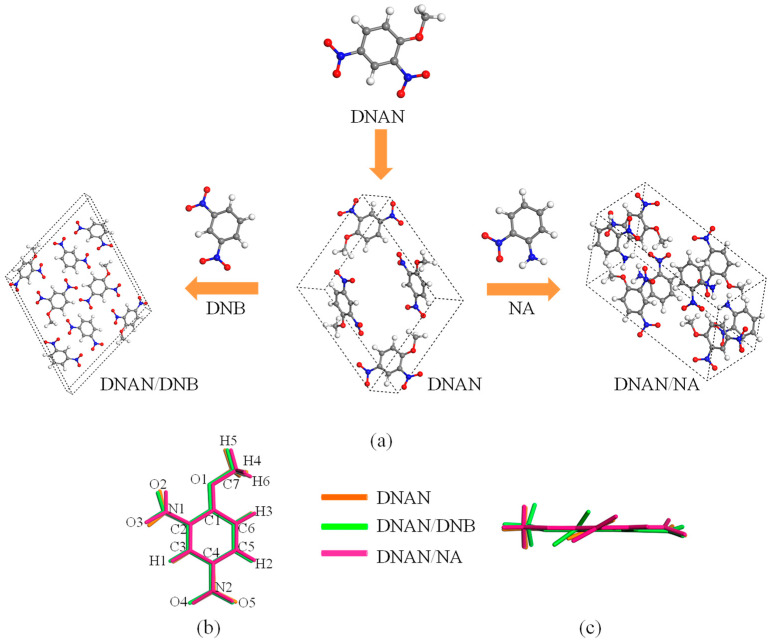
Crystal and molecular structure (**a**) of DNAN, DNB, NA, DNAN/DNB, and DNAN/NA. Orange, green, and pink represent the DNAN molecules originating from pure DNAN crystals, DNAN/DNB co-crystals, and DNAN/NA co-crystals, respectively. (**b**) and (**c**) are front and side views, respectively.

Figure 1a displays the molecular components and unit cell of the three DNAN-based crystals. The crystal structures taken from the CCDC database were fully relaxed. According to the experimental method described by the authors of [10], DNAN was mixed with DNB or NA at the molar ratio of 1:1 to synthesize the two cocrystals. Both DNAN and DNB include two nitro groups, while NA has one nitro and one amino. The three crystals are all *P*2_1_/n space group with cell parameter of Z = 4. Their calculated lattice parameters, total energy, and coordinates were listed in Table 1, Table S1 and Table S2 (Supplementary Materials), respectively. It is found that the calculation errors of the lattice parameters for all three crystals are within 2% compared with the experimental values [10], which proves the accuracy of our calculations. 

To analyze the effects of DNB and NA on the structure of DNAN, we superimposed the DNAN molecules from the three crystals. Structural changes were visually displayed in a front view (Figure 1b) and side view (Figure 1c). Table 2 shows the detailed changes of the bond lengths and torsion angles of the DNAN molecules in the three crystals. Generally, the torsion angles of nitro groups (C3-C4-N2-O5 and C3-C2-N1-O2) and alkoxy groups (C2-C1-O1-C7) of the DNAN molecules in the two cocrystals are smaller than those in the pure DNAN crystal. It suggests that cocrystallization forced the groups of the DNAN molecules to incline from a horizontal direction. Weak intermolecular interactions between DNAN and DNB or NA in the cocrystals may contribute to the transformation. More details of the interactions will be discussed below. In contrast with the transformation in the torsion angles, tiny changes were presented in the bond lengths. This may suggest that cocrystallization has little effect on the strength of chemical bonds. 

#### 2.1.2. Crystal Stacking 

**Figure 2:**
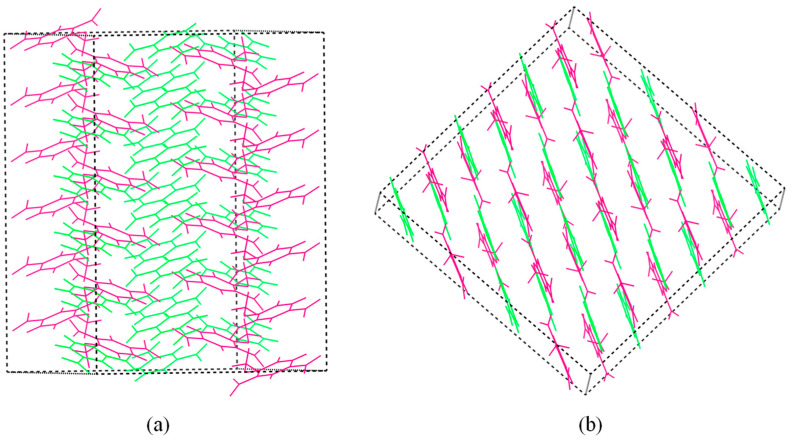
Crystal packing of the DNAN/DNB (**a**) and DNAN/NA (**b**) cocrystals. Pink and green represent DNAN and DNB or NA, respectively.

Two different crystal stacking models in the DNAN/DNB and DNAN/NA cocrystals were presented in Figure 2. When DNAN cocrystalizes with DNB, the cocrystal shows mix stacking (Figure 2a). Meanwhile, for the DNAN/NA cocrystal, it exhibits face-to-face stacking (Figure 2b). Previous studies suggested that face-to-face stacking can improve the stability as the interlayer sliding can disperse the external stimuli [14,15,16]. As confirmed in previous experimental studies, DNAN/NA shows distinguished stability among the DNAN-based cocrystals with impact sensitivity (*H*_50_) of >112 cm and spark sensitivity (*E*_50_) of 1.78 J [10]. On the contrary, *E*_50_ for DNAN/DNB is 0.85 J, which is as low as that of TNT. The significant different crystal stacking models may contribute to the sensitivity variation between the two cocrystals. 

### 2.2. Non-Covalent Interactions

**Figure 3:**
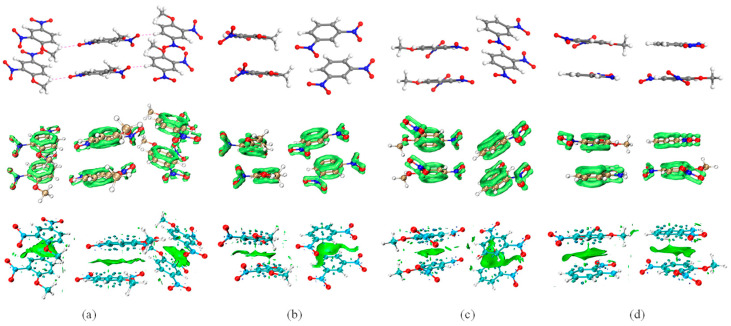
Interlayer interaction illustration of DNAN (**a**), DNAN/DNB (**b**,**c**) and DNAN/NA (**d**). Representative clusters in Ball-Stick models (**top** row), LOL-π (**middle** row), and IRI-π (**bottom** row). Bright green surface denotes the LOL-π isosurface (the isovalue is 0.5 a.u.) and IRI-π (the isovalue is 1.0 a.u.) in middle and bottom row, respectively.

**Figure 4:**
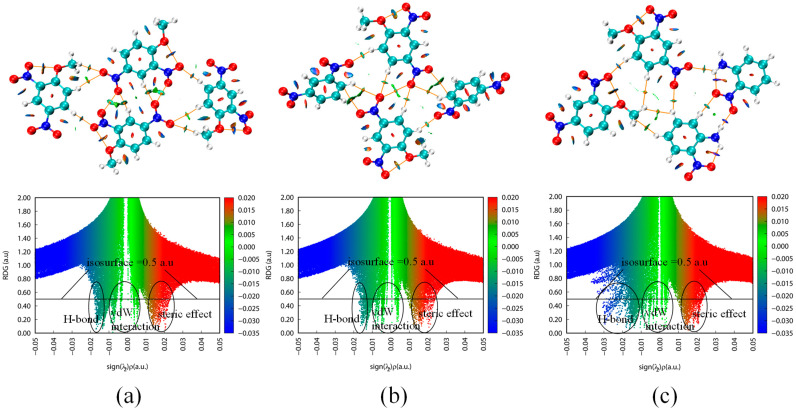
RDG isosurface diagrams (**top** row), scatter plot (the isovalue is 0.5 a.u., **bottom** row), and AIM analysis in the crystals DNAN (**a**), DNAN/DNB (**b**), and DNAN/NA (**c**). Blue, green, and red represent H-bond, vdW interaction, and steric effect, respectively. Orange circles and lines denote BCP and critical paths in the AIM analysis, respectively.

Non-covalent interactions (NCI) including hydrogen bond (H-bond), vdW interaction, and steric effect, play a non-negligible role in regulating the properties of energetic crystals [17,18]. Additionally, NCI in interlayer and intralayer may have different effects when modulating the properties of EM [14,19]. Here, an interaction region indictor (IRI)-π (Figure 3, bottom row) and reduced density ingredient (RDG) (Figure 4) were adopted to describe the weak intermolecular interactions of the interlayer and intralayer in the DNAN-based cocrystals. In order to analyze the interlayer interactions, clusters were selected from the optimized structures of the three crystals (Figure 3, top row). Specifically, as two kinds of binding model existed between DNAN and DNB (ether-methyl of DNAN towards or opposite the nitro group of DNB), two representative clusters were selected from the DNAN/DNB cocrystal (Figure 3b,c).

#### 2.2.1. Interlayer Interactions

Since there are the benzene rings and nitro groups in DNAN, DNB, or NA, π electrons should be presented in these clusters. Localized orbital locator (LOL)-π analysis (Figure 3, middle row) was performed beforehand to identify the interlayer interactions. If the occupation numbers of σ-occupied orbitals are set to zero and the occupation numbers of π-occupied orbitals are kept constant, the delocalization of electrons in the molecules can be obtained. As expected, the benzene rings of DNAN, DNB and NA exhibit big-π orbital delocalization, but DNB and NA present bigger and flatter π orbital delocalization due to their flat structures. Additionally, the nitro groups show π electrons delocalization, as well as the lone pair of electrons on the amino group. 

Two different crystal stacking models for DNAN/DNB and DNAN/NA were shown in Figure 2. The big π-π conjugation between the DNAN and NA molecules (Figure 3d, middle row) facilitates the DNAN/NA cocrystal to form the face-to-face stacking model (Figure 2b). However, the arrangement of the molecules in DNAN/DNB (Figure 3b,c) is distinct from that in DNAN/NA. Rather than layer stacking, the DNAN and DNB molecules are next to each other, forming a zigzag structure, resulting in the mix stacking model, as shown in Figure 2a. Hence, for the interlayer stacking of DNAN/DNB, DNAN interacts with DNAN, while DNB interacts with DNB (Figure 3b,c, top row). Based on π-electron density and its gradient [20], IRI-π analysis can be used to measure the interlayer interactions (Figure 3, bottom row), which are from the interactions between π electrons. To quantitatively characterize the IRI-π surface, the function of electron density was integrated (Table 3). The electron number of the domain can represent the strength of the interlayer interactions. As expected, the DNAN/NA cocrystal with the electron number of 0.033 between the DNAN and NA molecules shows the highest interlayer interactions among the three crystals. Furthermore, two kinds of interlayer electron distribution were identified in the DNAN/DNB cocrystal. The electron numbers between the DNB and DNB molecules are higher than those between the DNAN and DNAN molecules.

#### 2.2.2. Intralayer Interactions

Similar to IRI, RDG function calculated from the electron density gradient norm function can be also employed to reveal NCI among intralayer molecules [21]. To comprehensively illustrate the interaction style, the representative clusters including the intralayers were built from the three crystals. In Figure 4, RDG isosurfaces and scatter plots were used to visualize the intralayer NCI of the three crystals. Generally, NCI were identified in all three crystals. In the DNAN/NA cluster, H-bonds, which are denoted in blue, have wider distribution than those in the other two crystals. Additionally, the vdW interactions in the DNAN/DNB cocrystal are stronger than those in the pure DNAN crystal. 

The electron number and q_bind_ index of several representative interactions (Table 3) can quantitatively compare the intralayer NCIs in the three crystals. Based on λ_2_, the second-largest eigenvalue of the electron-density Hessian matrix [22], q_bind_ index can be obtained to describe the strength of the H-bond involved in intralayer intermolecular interactions. A more negative q_bind_ value implies stronger interactions. In the DNAN and DNAN/DNB crystals, the main type of intermolecular NCI is the H-bond of C-H⋯O, while the N-H⋯O H-bonds exist in DNAN/NA due to the existence of the amino in NA (Table 3, Figure 4 top row). Notably, the H-bond between the two NA molecules in the DNAN/NA cluster (N40-H41⋯O21) has a q_bind_ value of −1.56 and an electron number of 0.006, which are much higher than those of all other H-bonds in the representative clusters of the three crystals. Meanwhile, in Figure 4c, the blue RDG isosurface between the amino and nitro groups from the two NA molecules of DNAN/NA also confirms the highest NCI in the cocrystal of DNAN/NA. 

Since the crystal stacking style of DNAN/NA changes to the face-to-face one, the DNAN and NA molecules are closer to each other. As a result, in the DNAN/NA cluster, a stronger H-bond (C14-H15⋯O53) with a q_bind_ value of −0.83 was identified between the nitro group of NA and H of benzene from DNAN compared with the DNAN and DNAN/DNB clusters. Compared to the pure DNAN crystal, the DNAN/DNB cocrystal has more strong intralayer H-bonds, since the DNAN and DNB molecules are next to each other (Figure 4b, Table 3). 

Atom-in-molecule (AIM) theory based topology analysis provides the details of series of real space functions at the bond critical points (BCP) [23,24], such as electron density (ρ), Laplacian of electron density (▽^2^ρ), kinetic energy (G_BCP_), potential energy density (V_BCP_), and energy density (E_BCP_/H_BCP_). Based on these real space functions, the bonding strength, boding type, and boding energy can be quantitated and classified [25]. In Table 4 and Figure 4, the AIM analysis was applied to further get the intra- and inter-molecular interaction energies (E_int_) of NCI, especially the H-bonds. According to the equations provided by Mata et al. [26], both E_int1_ (0.429 G_BCP_) and E_int2_ (−0.5 V_BCP_) were calculated and compared, as shown in Table 4. Regarding to the intra-molecular interactions, the H-bonds of N-H⋯O in the NA molecule of the DNAN/NA cocrystal are significantly stronger than those of O⋯O in the pure DNAN or DNAN/DNB crystal. Furthermore, the H-bond N40-H41⋯O21 is the strongest inter-molecular interactions with E_int_ > 20 kJ/mol, consistent with the above q_bind_ analysis. This energy level is considered to be a strong H-bond according to the classification criteria suggested by Emamian et al. [25].

Based on the above interaction analysis, it is found that the DNAN/NA cocrystal possesses strong H-bonds and face-to-face crystal stacking formed by the π-π interactions. This may contribute to its relatively high impact and spark stability. However, because DNAN/DNB has a mix-stacking model and stronger inter- and intra-layer NCIs, it will be prone to being triggered by electric spark compared to the pure DNAN crystals. 

#### 2.2.3. ESP Analysis

**Figure 5:**
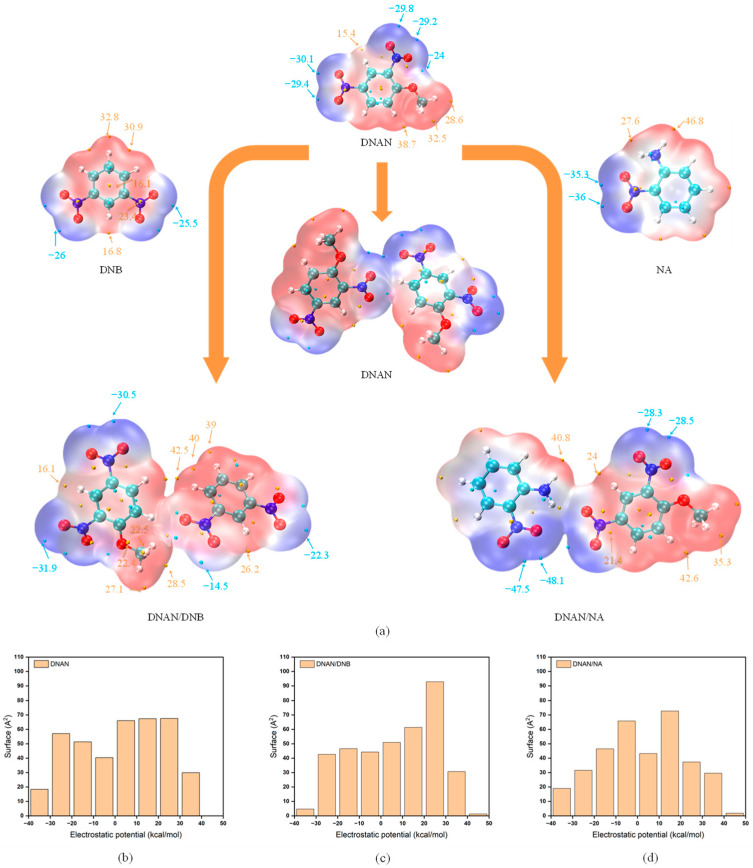
ESP in different clusters from pure DNAN, DNAN/DNB, and DNAN/NA (**a**), and surface−−area distribution of ESP of pure DNAN (**b**), DNAN/DNB (**c**), and DNAN/NA (**d**).

Electrostatic surface potential (ESP) is obtained based on electronic density, which has been widely used to quantitatively depict the nucleophilic and electrophilic sites [27]. In the DNAN-based crystals, the nitro and amino groups are characterized as nucleophilic and electrophilic sites, respectively (Figure 5a). Furthermore, the nitro group of the NA molecule has more negative potential than the nitro group of the DNAN or DNB molecule. This will lead to the variation of the ESP distribution in different crystals. As shown in Figure 5c and d, the ESPs of the two cocrystals distribute unevenly compared with the pure DNAN crystal (Figure 5b). The DNAN/DNB cocrystal has a more positive ESP range of 20–30 kJ/mol, whereas the DNAN/NA cocrystal has a more negative ESP range of −10–0 kJ/mol. As confirmed by the above NCI analysis, in the DNAN/DNB cocrystal, the nitro group of the DNB molecule will interact with H of the benzene and ether-methyl from the DNAN molecule, while for DNAN/NA, the amino group will interact with the nitro group from DNAN. 

### 2.3. Electronic Properties

**Figure 6:**
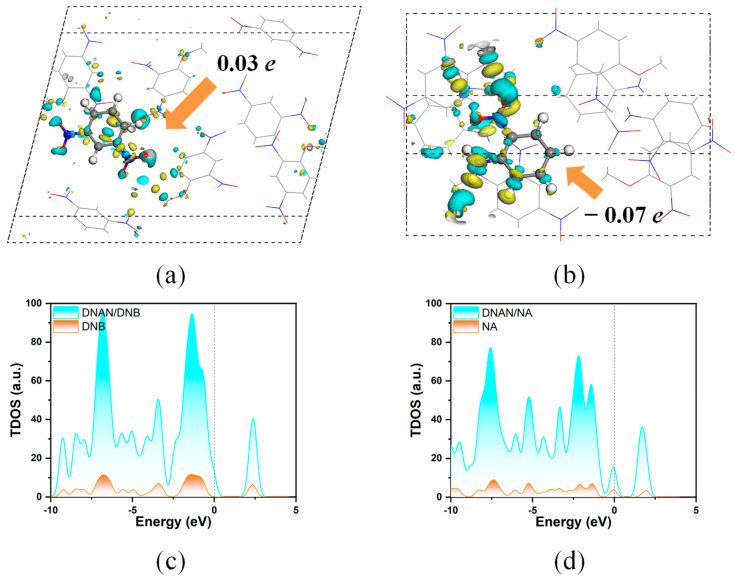
EDD (**Top** row) and TDOS (**bottom** row) in the two cocrystals: DNAN/DNB (**a**,**c**) and DNAN/NA (**b**,**d**). Blue and yellow represent the increase and decrease in the electron density, respectively (the isovalue is 0.01).

Electronic structures play a crucial role in disclosing elusive intermolecular interactions; thus, it is necessary to analyze the electronic properties in the DNAN-based cocrystals. To obtain more intuitive images of electron transformation, electronic density difference (EDD) was calculated, as presented in Figure 6a,b.

It is noteworthy that there is a more significant electron fluctuation around the NA molecules than around the DNB molecules, wherein blue and yellow stands for electron accumulation and consumption, respectively. Quantitatively, their corresponding charge transfers are also marked in inset by means of a Hirshfeld population analysis; that is, higher electron charge transformation is found to exist in DNAN/NA (0.07 e) compared with DNAN/DNB (0.03 e). Unexpectedly, these two coformers feature diverse electronic characteristics, indicating that DNAN is sensitive to these molecules, which is conducive to electron transfer. To better understand the interactions between them, the total density of state (TDOS) of the two cocrystals was presented in Figure 6c,d. The electrons can be transformed from the amino group of NA (Figure 6b) due to the strong electron-donor capacity of the amino group. This suggests there is higher electronic activity in the DNAN/NA cocrystal. Compared with NA, the TDOS of DNB shows that DNB has high orbital overlap with DNAN (Figure 6c) and a large energy gap around Fermi energy level. Additionally, a small peak is present in the DOS of NA for the Fermi energy (Figure 6d). This result is consistent with the EDD analysis above and demonstrates that DNAN/NA exhibits higher chemical activity compared to DNAN/DNB. 

**Figure 7:**
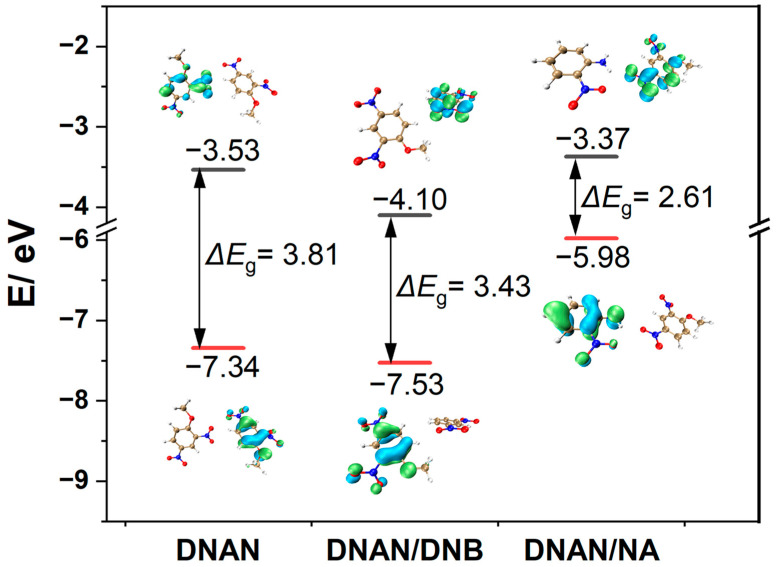
HOMO and LUMO and energies and ΔE_g_ of DNAN, DNAN/DNB, and DNAN/NA. The isovalue is 0.05 a.u.

In molecules, the most important orbitals that take part in the intermolecular interactions are the frontier molecular orbitals (FMO), including the highest occupied molecular orbital (HOMO) and lowest unoccupied molecular orbital (LUMO) [28]. The values of HOMO and LUMO and their energy gap (ΔE_g_) can reflect the chemical reaction activity. The electrons in the molecules with lower ΔE_g_ are easier to excite, which improves the activity of chemical reaction. The HOMO and LUMO energies and ΔE_g_ for representative clusters of DNAN, DNAN/DNB and DNAN/NA were calculated at the B3LYP level with 6-311G (d, p) basis set and are pictured in Figure 7. 

In Figure 7, green and blue represent the positive and negative isosurface, respectively. Generally, the band gaps of the two cocrystals decrease compared with the pure DNAN crystal (3.8 eV), which suggests that cocrystallization can increase chemical activity for DNAN. The HOMO energy of the DNAN/NA cocrystal is obviously elevated to −5.98 eV from −7.34 eV of the DNAN crystal or from −7.53 eV of the DNAN/DNB cocrystal. This can be attributed to the existence of strong electron donor group—the amino group in NA. Hence, DNAN/NA (ΔE_g_ = 2.6 eV) exhibits a relatively high molecular activity, consistent with the results of EDD and DOS discussed above (Figure 6).

### 2.4. Melting Process Simulations 

**Figure 8:**
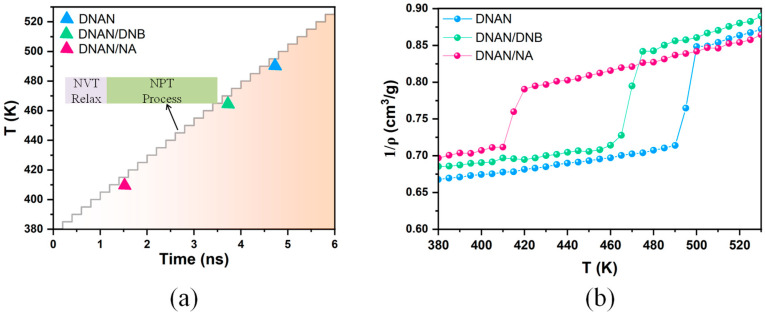
Schematic melting point simulation procedure (**a**) and inverse density vs. temperature for the three crystals (**b**). Blue, green, and pink represent DNAN, DNAN/DNB, and DNAN/NA, respectively. Insert view in (**a**) denotes the two stages for each temperature; purple and green represent 50 ps NVT relaxation and 150 ps NPT simulation, respectively.

Since *T*_m_ significantly determines the application of melt-cast explosive, we performed MD simulations to predict *T*_m_ of the DNAN-based cocrystals. The crystal structures were optimized by the COMPASS force field prior to MD simulations. The lattice parameters were compared with the experimental results. The accuracy of the COMPASS force field was validated, since the errors are within 5% (Table S3). Figure 8a displays schematic detail melting procedures. The simulation temperature range is from 380 K to 530 K, including *T*_m_, and the heating velocity is set to 5 K/stage. For each stage, after 50 ps NVT-MD relaxation, 150 ps NPT-MD simulations were performed to simulate the melting process (Figure 8a). The specific volume density is abruptly shifted at *T*_m_, so by calculating the specific volume density, the *T*_m_ values of the three crystals can be obtained (Figure 8b). Generally, for all three crystals, the specific volume densities can be separated into two distinct stages. During the solid phase, the specific volume density in the three crystals is around 0.7 cm^3^/g. With the temperature increase, the specific volume density also increases, as expected. Additionally, the rate of the enlargement of the specific volume density changes significantly at *T*_m_. In Figure 8b, the *T*_m_ values of the three crystals can be directly obtained in the decreasing order of 495 K, 470 K, and 415 K, respectively. The result suggests that *T*_m_ for the DNAN/NA and DNAN/DNB cocrystals reduces compared with the pure DNAN crystal. Similar to the results obtained by Sun et al. through a thermogravimetry–differential scanning calorimetry (TG-DSC) experiment, *T*_m_ for the DNAN-based cocrystals is distinctly lower than that for the pure DNAN crystal [10]. The similar pattern proves the accuracy of our MD simulations. In addition, compared with the decomposition temperature (*T*_deco_) tested by TG-DSC [10], the temperature differences (Δ*T*) between *T*_m_ and *T*_deco_ were obtained to be 188 K for DNAN/NA, 152 K for DNAN/DNB, and 98 K for pure DNAN. With the biggest Δ*T*, DNAN/NA is suggested to be the most valuable DNAN-based melt-cast explosive. 

**Figure 9:**
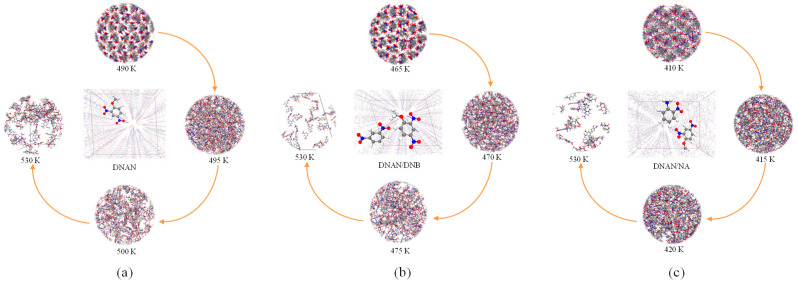
Snapshots of the melting processes of DNAN (**a**), DNAN/DNB (**b**), and DNAN/NA (**c**). Each perspective view in the middle denotes the representative molecular structure of each crystal.

As the crystals are heated, their structure, density, and phase change, allowing for visual observation of their melting processes. Figure 9 shows the snapshots of the melting processes of the three crystals. The regular arrangement of the crystals can be observed at the initial stage in Figure 9 (top snapshot). This suggests that all the systems are solid and are regularly arranged at the beginning of the melting progress. As the temperature increases, the crystals gradually dissolve and the morphologies become disorderly (Figure 9, right and bottom snapshot). By the end of our simulations at 530 K, the three crystals are all totally dissolved to liquid phase (Figure 9, left snapshot). The DNAN/NA cocrystal is the first one to begin to melt (Figure 9c), followed by the DNAN/DNB cocrystal (Figure 9b) and pure DNAN crystal (Figure 9a), which confirms that the melting point calculations in Figure 8b are reliable. As identified in the above electronic properties analysis, high molecular activity may contribute to the easiest phase change of DNAN/NA.

## 3. Materials and Methods

The original crystal structures of pure β-DNAN crystal (ID: 1041033), DNAN/DNB (ID: 1900909) and DNAN/NA (ID: 1900901) cocrystals were derived from Cambridge Crystallographic Data Centre (CCDC). DFT calculations were performed with ultrasoft pseudopotentials and plane-wave expansion of the wave functions, as implemented in the CASTEP module [29]. Crystal structures were relaxed by the Broyden, Fletcher, Goldfarb, and Shanon (BFGS) method [30]. The Perdew–Burke–Ernzerhof (PBE) functional of generalized gradient approximation (GGA) was used to calculate the electronic exchange-correlation. Grimme’s method was employed to calculate dispersion correction [31]. The cut-off energy of plane-wave is 340 eV. The Monhkorst–Pack scheme was used to implement brillouin zone sampling with k-point grid of 1 × 1 × 1.

In order to compare intermolecular interactions and electronic properties between the three crystals, a series of quantum chemical approaches were adopted, as shown below. Representative clusters of the three crystals were built from the above relaxed crystal structures. Their electron wave functions were calculated by the hybrid DFT-B3LYP method with 6-311G (d, p) basis set.

Localized orbital locator (LOL) was firstly defined by Schmider and Becke in 2000 to characterize the localization of electron pairs [32], and can be calculated according to Equation (1). The τ represents the local kinetic energy densities. It can be inferred that the γ(r) represents the ratio of the uniform electron pneumatic energy density to the current actual system kinetic energy density. The smaller the γ(r), the stronger the localization of electron pairs. LOL-π provided by Gonthier et al. [33], which calculated LOL based on π electrons, can be used to decipher the π interactions. The intuitive expression of LOL-π can be illustrated using Multiwfn software and visual molecular dynamics (VMD) [34].

(1) $$\gamma \left(r\right)={\;\tau }_{\sigma }^{\mathrm{LSDA}}/\tau_{\sigma }^{\mathrm{exact}}/\left(1+\tau_{\sigma }^{\mathrm{LSDA}}/\tau_{\sigma }^{\mathrm{exact}}\right)$$

Interaction region indictor (IRI) was originally introduced by Tian and Chen to describe interactions that can include both chemical bond and non-covalent interactions (NCI) [20]. Equation (2) can be used to calculate IRI. Based on the delocalization information of π electrons through the above LOL-π calculations, IRI-π can be used to reveal the π interaction strength.

(2) $$\mathrm{IRI}\left(r\right)=\left|▽\rho \left(r\right)\right|/{\left[\rho \left(r\right)\right]}^{a}$$

Reduced density ingredient (RDG) firstly described by Yang et al. [21] can be used to detect NCI in real space. RDG can be calculated based on the electron density and its derivatives (Equation (3)), the dimensionless form of electron density gradient norm function.

(3) $$\mathrm{RDG}\left(r\right)=\frac{1}{2{\left(3\pi^{2}\right)}^{1/3}}\frac{\left|▽\rho \left(r\right)\right|}{\rho {\left(r\right)}^{4/3}}$$

The atom-in-molecule (AIM) theory proposed by Bader [23] can be used to analyze electron density (ρ). In the AIM theory, the value of ρ and the sign of ▽^2^ρ at bond critical point (BCP) can reflect bonding strength and type [35]. 

Electrostatic surface potential (ESP, V(r)), expressed as Equation (4), can be used to describe chemical reactivity and molecular interactive behavior [27]. ESP is in terms of the electronic density (ρ(r)) and the charges (Z_A_) and positions (R_A_) of the nuclei.

(4) $$V\left(r\right)=\underset{A}{\sum }\frac{Z_{A}}{\left|R_{A}-r\right|}-\int \frac{\rho \left(r^{’}\right){\mathrm{dr}}^{’}}{\left|r^{’}-r\right|}$$

The highest occupied molecular orbital (HOMO) and lowest unoccupied molecular orbital (LUMO) were computed by Multiwfn 3.8 software [24] and their plots were outputted through VMD [36]. Intermolecular interaction energy (E_int_) was calculated based on the electron density (q_BCP_). The kinetic energy density (G_BCP_) (Equation (5)) and potential energy density (V_BCP_) (Equation (6)) at the BCP were calculated using the following equations in previous reports [26]:

E_int1_ = 0.429 × G_BCP_ (5)

E_int2_ = −0.5 × V_BCP_ (6)

The electronic density difference (EDD), Hirshfeld charge population, and density of state (DOS) of the DNAN/DNB and DNAN/NA cocrystals were calculated through the CASTEP module [37] in Materials Studio software. Detail codes were showed in the Supporting Information (Code S1). For the EDD analysis, the isovalue was set as 0.01. The corresponding single molecule was selected for the total DOS (TDOS) analysis of DNB or NA. 

To predict melting temperature *T*_m_, MD simulations were performed by the Forcite module. Firstly, structure optimizations were carried out by the COMPASS force field [38] with ESP charge assigned to the atoms. Then, 10 × 3 × 3, 2 × 12 × 2 and 6 × 2 × 4 supercells for the pure DNAN, DNAN/DNB cocrystal and DNAN/NA cocrystal, respectively, were used to perform MD simulations. Here, a temperature range of 380 K to 530 K covering the melting points was applied to simulate the melting process with the heating velocity as 5 K/stage. For each stage, after 50 ps NVT relaxation, 150 ps NPT simulations were performed with a time step of 1 fs. Optimized *Perl* scripts (Code S2) were used to execute these procedures in Materials Studio.

## 4. Conclusions 

Our studies analyze the properties of the DNAN crystal under the influence of two different coformers, DNB and NA. With face-to-face stacking conformation and strong interlayer interactions, DNAN/NA shows relatively low impact sensitivity. Due to the strong electron-donor ability of the amino group in NA, there are more electrons transformed in the DNAN/NA cocrystal, as evidenced by the lower DOS overlap and smaller HOMO-LUMO energy gap. Meanwhile, the melting points of the three DNAN-based crystals were predicted by MD. Through cocrystallization, their melting points were significantly decreased compared with the pure DNAN crystal. Higher molecular activity may contribute to the decrease in *T*_m_ for DNAN/NA. In conclusion, different components can significantly influence the chemical and physical properties of the cocrystal explosives. Our melt point predictions by MD provide a wide view of the application of the melt-cast explosives. These insights may guide further design and synthesis of melt-cast cocrystal explosives. 

## Figures and Tables

**Table 1:** Calculated and experimental lattice parameters of the pure DNAN crystal and its cocrystals.

		DNAN	DNAN/DNB	DNAN/NA
Space group		*P*2_1_/n	*P*2_1_/n	*P*2_1_/n
	This work	3.88	18.86	7.58
a (Å)	Expt. [10]	3.91	18.81	7.43
	Error (%)	0.77%	−0.26%	−2.02%
	This work	13.54	3.79	17.55
b (Å)	Expt. [10]	13.78	3.87	17.57
	Error (%)	1.74%	2.07%	0.11%
	This work	15.32	21.82	11.49
c (Å)	Expt. [10]	15.42	21.91	11.49
	Error (%)	0.65%	0.41%	0%
	This work	90.00	90.00	90.00
α (°)	Expt. [10]	90.00	90.00	90.00
	Error (%)	0%	0%	0%
	This work	95.30	106.70	95.22
β (°)	Expt. [10]	95.31	106.70	95.21
	Error (%)	0.01%	0%	−0.01%
	This work	90.00	90.00	90.00
γ (°)	Expt. [10]	90.00	90.00	90.00
	Error (%)	0%	0%	0%

**Table 2:** Representative bond lengths and torsion angles in the three crystals.

	DNAN	DNAN/DNB	DNAN/NA
Bond length (Å)			
C2-NO_2_	1.459	1.456	1.451
C4-NO_2_	1.45	1.45	1.448
C1-OCH_3_	1.337	1.335	1.337
O1-CH_3_	1.449	1.458	1.446
Torsion angles (°)			
C3-C4-N2-O5	−169.7	−169.1	−162.3
C3-C2-N1-O2	−156.9	−139.2	−150.2
C2-C1-O1-C7	179.1	171.2	177.7

**Table 3:** List of electron numbers (a.u.) and q_bind_ (a.u.) of interlayer weak interactions and intralayer intermolecular weak interactions of the three crystals.

Type	Crystal	Interactions	Electron Number × 10	q_bind_ × 10^3^
Interlayer	DNAN	DNAN-DNAN	0.21	0.05
	DNAN/DNB	DNAN-DNAN	0.21	0.32
		DNB-DNB	0.26	0.02
	DNAN/NA	DNAN-NA	0.33	0.45
Intralayer	DNAN	C10-H3⋯O40	0.02	−0.43
		C6-H7⋯O57	0.03	−0.67
		C68-H69⋯O60	0.01	0
		C66-H67⋯O37	0.03	−0.68
		C70-H73⋯O60	0.02	−0.43
		C8-H9⋯O40	0.01	0
	DNAN/DNB	C8-H9⋯O40	0.02	−0.36
		C48-H49⋯O54	0.03	−0.33
		C32-H33⋯O2	0.03	−0.33
		C10-H11⋯O53	0.01	−0.26
		C62-H63⋯O1	0.01	−0.27
		C69-H70⋯O24	0.02	−0.36
	DNAN/NA	N24-H26⋯O3	0.02	−0.43
		C14-H15⋯O53	0.03	−0.86
		C48-H49⋯O4	0.02	−0.36
		C50-H51⋯O22	0.02	−0.53
		N40-H41⋯O21	0.06	−1.56
		C48-H49⋯H70	0.01	−0.01
		C46-H47⋯H72	0.03	−0.42

**Table 4:** Topological parameters of the three DNAN-based crystals at the (3, −1) critical points: electron densities (ρ, e Å^−3^), Laplacian of electron densities (▽^2^ρ, e Å^−5^), Laplacian of kinetic energy (G_BCP_, a.u.), potential energy density (V_BCP_, a.u.), energy density (E_BCP_/H_BCP_, a.u.), and interaction energy (E_int_, kJ/mol). For each crystal, the interactions are classified into two types as intra-molecular and inter-molecular interactions.

Crystal	Type	Interaction	ρ	▽^2^ρ	G_BCP_	V_BCP_	E_BCP_/H_BCP_	E_int1_	E_int2_
DNAN	Intra- molecular	O17-O16	0.017	0.070	0.016	−0.014	0.002	17.59	17.95
		O56-O57	0.017	0.070	0.016	−0.014	0.002	17.49	17.74
		O76-O77	0.017	0.070	0.016	−0.014	0.002	17.59	17.95
		O36-O37	0.017	0.070	0.016	−0.014	0.002	17.49	17.74
	Inter- molecular	C10-H3⋯O40	0.009	0.034	0.007	−0.006	0.001	7.95	7.44
		C8-H9⋯O40	0.004	0.013	0.003	−0.002	0.001	3.16	2.95
		C6-H7⋯O57	0.013	0.042	0.009	−0.007	0.002	9.97	9.57
		O58-O39	0.007	0.027	0.006	−0.005	0.001	6.83	7.09
		C43-H44⋯O39	0.008	0.029	0.006	−0.005	0.001	6.89	6.46
		C23-H24⋯O59	0.008	0.029	0.006	−0.005	0.001	6.89	6.46
		O38-O59	0.007	0.027	0.006	−0.005	0.001	6.83	7.09
		C68-H69⋯O60	0.004	0.013	0.003	−0.002	0.001	3.16	2.95
		C70-H73⋯O60	0.009	0.034	0.007	−0.006	0.001	7.96	7.45
		C66-H67⋯O37	0.013	0.042	0.009	−0.007	0.002	9.96	9.57
DNAN/ DNB	Intra- molecular	O1-O5	0.013	0.052	0.012	−0.010	0.001	13.27	13.78
		O57-O53	0.013	0.052	0.012	−0.010	0.001	13.27	13.78
	Inter- molecular	C8-H9⋯O40	0.008	0.031	0.006	−0.005	0.001	7.31	6.85
		O2-C32	0.005	0.017	0.003	−0.003	0.001	3.91	3.49
		C10-H11⋯O53	0.007	0.027	0.006	−0.004	0.001	6.28	5.78
		C32-H33⋯O2	0.004	0.013	0.003	−0.002	0.001	3.17	2.96
		O1-O53	0.005	0.019	0.004	−0.003	0.001	4.52	4.33
		C48-H49⋯O54	0.004	0.013	0.003	−0.002	0.001	3.17	2.96
		C62-H63⋯O1	0.007	0.027	0.006	−0.004	0.001	6.28	5.78
		C48-O54	0.005	0.017	0.003	−0.003	0.001	3.91	3.49
		C69-H70⋯O24	0.008	0.031	0.006	−0.005	0.001	7.31	6.85
DNAN/ NA	Intra- molecular	N40-H42⋯O38	0.030	0.116	0.026	−0.024	0.003	29.74	31.36
		O53-O57	0.016	0.066	0.015	−0.013	0.002	16.64	17.04
		N24-H26⋯O22	0.029	0.115	0.026	−0.023	0.003	29.44	30.75
		O1-O5	0.016	0.067	0.015	−0.013	0.002	16.72	17.09
	Inter- molecular	C46-H47⋯H72	0.006	0.021	0.004	−0.003	0.001	4.83	4.26
		N40-H41⋯O21	0.023	0.085	0.019	−0.016	0.002	21.23	21.49
		C48-H49⋯H70	0.004	0.012	0.002	−0.002	0.001	2.66	2.40
		C50-H51⋯O22	0.011	0.033	0.007	−0.006	0.001	8.01	7.97
		C48-H49⋯O4	0.008	0.031	0.006	−0.005	0.001	7.30	6.88
		C12-H13⋯H70	0.002	0.007	0.001	−0.001	0.000	1.50	1.25
		C14-H15⋯O53	0.015	0.061	0.012	−0.010	0.003	14.04	12.79
		N24-H26⋯O3	0.010	0.037	0.008	−0.006	0.002	8.80	8.22

## Data Availability

The data presented in this study are available on request from the corresponding author.

## Supplementary Materials

The following supporting information can be downloaded at: https://www.mdpi.com/article/10.3390/molecules27249010/s1, Table S1: The optimized total energies of three crystals; Table S2: The optimized coordinates of three crystals; Table S3: Lattice parameters comparison between optimized structures under COMPASS force field and experimental data; Code S1: Codes for calculating the electronic structures; Code S2: Codes for calculating *T*_m_.

## Sample Availability

Samples of the compounds are not available from the authors.
