# Supplementary material for: Effects of Cocrystallization on the Structure and Properties of Melt-Cast Explosive 2,4-Dinitroanisole: A Computational Study
Source: Molecules. 2022 Dec 17;27(24):9010. doi: 10.3390/molecules27249010 (PMC9783160; doi:10.3390/molecules27249010)
Supplement: Supplementary file 1 [file molecules-27-09010-s001.zip › molecules-2061836-supplementary.pdf]

Supporting Information

Table S1. The optimized total energies of three crystals

	**pure DNAN**	**DNAN/DNB**	**DNAN/NA**
Total energy (eV)	-15599.16	-28705.04	-25357.16

Table S2. The optimized coordinates of three crystals

**Item**	**pure DNAN**	**DNAN/DNB**	**DNAN/NA**
Molecule Structure	80 80 0 0 0<br>SMALL<br>USER_CHARGES	144 144 0 0 0<br>SMALL<br>USER_CHARGES	144 144 0 0 0<br>SMALL<br>USER_CHARGES
Atom	1 C1 1.52246 4.35193 14.1535 C.3 0 **** 0	1 O1 9.03122 2.9782 9.58905 O.3 0 **** 0	1 O1 1.90829 11.674 5.05163 O.3 0 **** 0
	2 C2 1.85137 3.33152 15.0941 C.3 0 **** 0	2 O2 9.72386 1.72731 11.2862 O.3 0 **** 0	2 O2 2.51776 13.5322 6.09854 O.3 0 **** 0
	3 C3 2.37227 2.11712 14.6734 C.3 0 **** 0	3 O3 14.6589 1.42981 11.7542 O.3 0 **** 0	3 N1 2.48858 12.2599 6.02656 N.3 0 **** 0
	4 H1 2.62342 1.35891 15.4113 H 0 **** 0	4 O4 15.4151 0.253509 10.0271 O.3 0 **** 0	4 N2 3.90688 13.4071 8.35268 N.3 0 **** 0
	5 C4 2.56483 1.89495 13.3166 C.3 0 **** 0	5 O5 14.9853 1.88637 7.87236 O.3 0 **** 0	5 H2A 4.44451 13.7514 9.16323 H 0 **** 0
	6 C5 2.27247 2.87896 12.3665 C.3 0 **** 0	6 N1 9.91986 2.31741 10.1905 N.3 0 **** 0	6 H2B 3.55576 14.0601 7.64473 H 0 **** 0
	7 H2 2.44396 2.69435 11.3058 H 0 **** 0	7 N2 14.6088 1.06268 10.5528 N.3 0 **** 0	7 C1 3.08989 11.482 7.04664 C.3 0 **** 0
	8 C6 1.75961 4.092 12.7885 C.3 0 **** 0	8 C1 12.6736 2.57403 7.69012 C.3 0 **** 0	8 C2 3.01391 10.0784 6.92471 C.3 0 **** 0
	9 H3 1.52615 4.84837 12.0416 H 0 **** 0	9 H1 12.8124 2.91668 6.66814 H 0 **** 0	9 H2 2.51794 9.66942 6.0497 H 0 **** 0
	10 C7 0.731579 6.55955 13.6438 C.3 0 **** 0	10 C2 11.4256 2.68002 8.27597 C.3 0 **** 0	10 C3 3.53511 9.25344 7.89222 C.3 0 **** 0
	11 H5 0.300768 7.36949 14.2384 H 0 **** 0	11 H2 10.5873 3.10093 7.72503 H 0 **** 0	11 H3 3.44948 8.17411 7.77915 H 0 **** 0
	12 H4 0.00327798 6.20457 12.9 H 0 **** 0	12 C3 11.2351 2.23086 9.58699 C.3 0 **** 0	12 C4 4.14881 9.82674 9.02715 C.3 0 **** 0
	13 H6 1.64814 6.89568 13.1431 H 0 **** 0	13 C4 12.2883 1.70796 10.3295 C.3 0 **** 0	13 H4 4.56072 9.18965 9.81007 H 0 **** 0

14 N1 1.67424 3.49718 16.5327 N.3 0 **** 0	14 H4 12.147 1.39673 11.3627 H 0 **** 0	14 C5 4.25035 11.1912 9.16286 C.3 0 **** 0
15 N2 3.09233 0.611551 12.8931 N.3 0 **** 0	15 C5 13.5324 1.59632 9.73022 C.3 0 **** 0	15 H5 4.74644 11.6306 10.0293 H 0 **** 0
16 O1 1.0314 5.51232 14.5998 O.3 0 **** 0	16 C6 13.7599 2.01117 8.38772 C.3 0 **** 0	16 C6 3.74452 12.082 8.17411 C.3 0 **** 0
17 O2 0.840483 4.34873 16.9397 O.3 0 **** 0	17 C7 15.258 2.4884 6.57293 C.3 0 **** 0	17 O3 5.26328 11.9216 4.84669 O.3 0 **** 0
18 O3 2.35231 2.76834 17.2971 O.3 0 **** 0	18 H7A 14.6668 1.99456 5.78868 H 0 **** 0	18 O4 6.22167 9.95417 4.49408 O.3 0 **** 0
19 O4 3.16711 -0.312281 13.7435 O.3 0 **** 0	19 H7B 16.325 2.32977 6.40029 H 0 **** 0	19 O5 7.255 7.6108 8.64107 O.3 0 **** 0
20 O5 3.45918 0.487192 11.6924 O.3 0 **** 0	20 H7C 15.034 3.56134 6.60691 H 0 **** 0	20 O6 8.70514 8.64848 9.96892 O.3 0 **** 0
21 C1 0.414129 11.2425 8.79557 C.3 0 **** 0	21 O6 15.7933 3.91106 3.87513 O.3 0 **** 0	21 O7 6.76391 13.4438 6.39671 O.3 0 **** 0
22 C2 0.0934235 10.2193 7.8555 C.3 0 **** 0	22 O7 17.6853 2.80444 4.23079 O.3 0 **** 0	22 N3 6.02392 10.9824 5.19225 N.3 0 **** 0
23 C3 -0.418443 9.0014 8.27599 C.3 0 **** 0	23 O8 13.6862 3.77729 -0.421284 O.3 0 **** 0	23 N4 7.8635 8.64392 9.03079 N.3 0 **** 0
24 H1 -0.659906 8.24006 7.53837 H 0 **** 0	24 O9 14.5745 2.59337 -2.07387 O.3 0 **** 0	24 C7 6.68442 11.0693 6.48171 C.3 0 **** 0
25 C4 -0.61624 8.78112 9.63238 C.3 0 **** 0	25 N3 16.7459 3.18677 3.49221 N.3 0 **** 0	25 C8 6.96782 9.87094 7.11832 C.3 0 **** 0
26 C5 -0.330769 9.76751 10.5822 C.3 0 **** 0	26 N4 14.5853 3.03963 -0.898662 N.3 0 **** 0	26 H8 6.71945 8.92836 6.63747 H 0 **** 0
27 H2 -0.504642 9.583 11.6427 H 0 **** 0	27 C8 16.7669 2.77039 2.0849 C.3 0 **** 0	27 C9 7.583 9.89716 8.36187 C.3 0 **** 0
28 C6 0.179475 10.9818 10.1609 C.3 0 **** 0	28 C9 17.8911 2.11488 1.58021 C.3 0 **** 0	28 C10 7.93205 11.1062 8.96976 C.3 0 **** 0
29 H3 0.410194 11.7391 10.9077 H 0 **** 0	29 H9 18.7426 1.91796 2.22849 H 0 **** 0	29 H10 8.39557 11.1057 9.95389 H 0 **** 0
30 C7 1.2041 13.4514 9.30232 C.3 0 **** 0	30 C10 17.9123 1.75355 0.235535 C.3 0 **** 0	30 C11 7.65713 12.298 8.3266 C.3 0 **** 0
31 H5 1.63217 14.2616 8.70624 H 0 **** 0	31 H10 18.7827 1.24429 -0.173475 H 0 **** 0	31 H11 7.93822 13.2313 8.8037 H 0 **** 0
32 H4 1.93657 13.0947 10.0414 H 0 **** 0	32 C11 16.8292 2.04459 -0.590304 C.3 0 **** 0	32 C12 7.02391 12.3195 7.0711 C.3 0 **** 0
33 H6 0.291317 13.7884 9.80912 H 0 **** 0	33 H11 16.8486 1.78635 -1.64693 H 0 **** 0	33 C13 7.15841 14.7061 6.98189 C.3 0 **** 0
34 N1 0.270037 10.3854 6.41685 N.3 0 **** 0	34 C12 15.723 2.69758 -0.0411949 C.3 0 **** 0	34 H13A 6.82361 15.4651 6.2664 H 0 **** 0
35 N2 -1.14508 7.49847 10.0553 N.3 0 **** 0	35 C13 15.6661 3.07184 1.29585 C.3 0 **** 0	35 H13B 8.25164 14.7469 7.09635 H 0 **** 0
36 O1 0.897726 12.4054 8.34731 O.3 0 **** 0	36 H13 14.802 3.583 1.71027 H 0 **** 0	36 H13C 6.65842 14.8451 7.95267 H 0 **** 0
37 O2 1.1042 11.2366 6.00966 O.3 0 **** 0	37 O1 17.9974 1.03897 15.1656 O.3 0 **** 0	37 O1 1.79137 2.89571 11.8501 O.3 0 **** 0
38 O3 -0.409578 9.65739 5.65323 O.3 0 **** 0	38 O2 17.3042 -0.212475 13.4694 O.3 0 **** 0	38 O2 1.18964 4.75139 10.7929 O.3 0 **** 0
39 O4 -1.22667 6.57685 9.20362 O.3 0 **** 0	39 O3 12.3667 -0.511438 13.0011 O.3 0 **** 0	39 N1 1.21431 3.47941 10.872 N.3 0 **** 0

40 O5 -1.5071 7.37338 11.2577 O.3 0 **** 0	40 O4 11.614 -1.68723 14.7299 O.3 0 **** 0	40 N2 -0.205778 4.62285 8.54511 N.3 0 **** 0
41 C1 2.36877 9.42807 0.904804 C.3 0 **** 0	41 O5 12.044 -0.0521494 16.883 O.3 0 **** 0	41 H2A -0.745188 4.9655 7.73502 H 0 **** 0
42 C2 2.03987 10.4485 -0.0357517 C.3 0 **** 0	42 N1 17.108 0.379365 14.5641 N.3 0 **** 0	42 H2B 0.1452 5.27743 9.25164 H 0 **** 0
43 C3 1.51896 11.6629 0.384894 C.3 0 **** 0	43 N2 12.4187 -0.877198 14.2028 N.3 0 **** 0	43 C1 0.612061 2.69964 9.85454 C.3 0 **** 0
44 H1 1.26781 12.4211 -0.352988 H 0 **** 0	44 C1 14.3531 0.643462 17.0617 C.3 0 **** 0	44 C2 0.688965 1.29619 9.97905 C.3 0 **** 0
45 C4 1.3264 11.8851 1.74171 C.3 0 **** 0	45 H1 14.2144 0.986372 18.0836 H 0 **** 0	45 H2 1.18683 0.888664 10.8536 H 0 **** 0
46 C5 1.61876 10.901 2.69179 C.3 0 **** 0	46 C2 15.601 0.748177 16.476 C.3 0 **** 0	46 C3 0.167284 0.469668 9.01349 C.3 0 **** 0
47 H2 1.44727 11.0856 3.75251 H 0 **** 0	47 H2 16.4391 1.171 17.0256 H 0 **** 0	47 H3 0.255062 -0.609342 9.12775 H 0 **** 0
48 C6 2.13162 9.688 2.26985 C.3 0 **** 0	48 C3 15.792 0.295435 15.1663 C.3 0 **** 0	48 C4 -0.448812 1.04131 7.87932 C.3 0 **** 0
49 H3 2.36508 8.93163 3.0167 H 0 **** 0	49 C4 14.7392 -0.228574 14.4239 C.3 0 **** 0	49 H4 -0.862333 0.402791 7.09867 H 0 **** 0
50 C7 3.15964 7.22044 1.41452 C.3 0 **** 0	50 H4 14.8806 -0.539762 13.3907 H 0 **** 0	50 C5 -0.551121 2.40535 7.74158 C.3 0 **** 0
51 H5 3.59046 6.41051 0.819948 H 0 **** 0	51 C5 13.4955 -0.341544 15.0238 C.3 0 **** 0	51 H5 -1.04804 2.84295 6.87455 H 0 **** 0
52 H4 3.88795 7.57542 2.15838 H 0 **** 0	52 C6 13.2683 0.0756605 16.3656 C.3 0 **** 0	52 C6 -0.0429205 3.29819 8.72656 C.3 0 **** 0
53 H6 2.24308 6.88432 1.91517 H 0 **** 0	53 C7 11.7724 0.551477 18.1819 C.3 0 **** 0	53 O3 5.82759 3.13275 11.3852 O.3 0 **** 0
54 N1 2.21701 10.2829 -1.47434 N.3 0 **** 0	54 H7A 12.3625 0.0566291 18.9664 H 0 **** 0	54 O4 4.87428 1.16266 11.7343 O.3 0 **** 0
55 N2 0.798903 13.1684 2.1653 N.3 0 **** 0	55 H7B 10.7051 0.395382 18.3552 H 0 **** 0	55 O5 3.83142 -1.17903 7.58682 O.3 0 **** 0
56 O1 2.85982 8.26768 0.458492 O.3 0 **** 0	56 H7C 11.999 1.62386 18.1477 H 0 **** 0	56 O6 2.39769 -0.134452 6.24646 O.3 0 **** 0
57 O2 3.05083 9.4313 -1.88133 O.3 0 **** 0	57 O6 11.2383 1.97629 -1.03382 O.3 0 **** 0	57 O7 4.33371 4.65711 9.82581 O.3 0 **** 0
58 O3 1.53896 11.0116 -2.23876 O.3 0 **** 0	58 O7 9.3506 0.862467 -1.3903 O.3 0 **** 0	58 N3 5.06912 2.19252 11.0375 N.3 0 **** 0
59 O4 0.724137 14.0923 1.31482 O.3 0 **** 0	59 O8 13.3466 1.83593 3.26339 O.3 0 **** 0	59 N4 3.22925 -0.143509 7.19374 N.3 0 **** 0
60 O5 0.432044 13.2928 3.36592 O.3 0 **** 0	60 O9 12.4496 0.664501 4.91997 O.3 0 **** 0	60 C7 4.40769 2.28016 9.74839 C.3 0 **** 0
61 C1 3.47709 2.53746 6.26277 C.3 0 **** 0	61 N3 10.2866 1.25095 -0.650643 N.3 0 **** 0	61 C8 4.12467 1.0816 9.11057 C.3 0 **** 0
62 C2 3.7978 3.56068 7.20283 C.3 0 **** 0	62 N4 12.4431 1.10488 3.74248 N.3 0 **** 0	62 H8 4.37497 0.138833 9.58989 H 0 **** 0
63 C3 4.30967 4.7786 6.78235 C.3 0 **** 0	63 C8 10.2632 0.839304 0.757842 C.3 0 **** 0	63 C9 3.50743 1.10773 7.86783 C.3 0 **** 0
64 H1 4.55114 5.53994 7.51995 H 0 **** 0	64 C9 9.13907 0.182516 1.26094 C.3 0 **** 0	64 C10 3.15909 2.31682 7.26016 C.3 0 **** 0
65 C4 4.50746 4.99887 5.42594 C.3 0 **** 0	65 H9 8.28858 -0.0155693 0.61167 H 0 **** 0	65 H10 2.69527 2.31675 6.27629 H 0 **** 0

66 C5 4.22198 4.01249 4.47612 C.3 0 **** 0	66 C10 9.11734 -0.181372 2.60483 C.3 0 **** 0	66 C11 3.43684 3.50848 7.90124 C.3 0 **** 0
67 H2 4.39586 4.19699 3.41567 H 0 **** 0	67 H10 8.24731 -0.691999 3.01292 H 0 **** 0	67 H11 3.1583 4.44092 7.42074 H 0 **** 0
68 C6 3.71174 2.79815 4.89748 C.3 0 **** 0	68 C11 10.1999 0.108683 3.43163 C.3 0 **** 0	68 C12 4.07036 3.53052 9.1567 C.3 0 **** 0
69 H3 3.48102 2.04093 4.15064 H 0 **** 0	69 H11 10.1804 -0.1524 4.48761 H 0 **** 0	69 C13 3.93982 5.91727 9.23432 C.3 0 **** 0
70 C7 2.68712 0.328611 5.75601 C.3 0 **** 0	70 C12 11.3052 0.764418 2.88428 C.3 0 **** 0	70 H13A 4.27277 6.67919 9.94749 H 0 **** 0
71 H5 2.25906 -0.481628 6.3521 H 0 **** 0	71 C13 11.3624 1.14213 1.54812 C.3 0 **** 0	71 H13B 2.84686 5.95751 9.11748 H 0 **** 0
72 H4 1.95465 0.685269 5.01696 H 0 **** 0	72 H13 12.2267 1.65395 1.13512 H 0 **** 0	72 H13C 4.44215 6.05243 8.26441 H 0 **** 0
73 H6 3.59991 -0.00839168 5.24922 H 0 **** 0	73 O1 8.98801 0.892099 6.9139 O.3 0 **** 0	73 O1 5.48802 5.89704 5.7654 O.3 0 **** 0
74 N1 3.62119 3.3946 8.64148 N.3 0 **** 0	74 O2 8.29537 2.14298 5.21673 O.3 0 **** 0	74 O2 4.87854 4.03874 4.71849 O.3 0 **** 0
75 N2 5.03631 6.28153 5.00308 N.3 0 **** 0	75 O3 3.36034 2.4405 4.74873 O.3 0 **** 0	75 N1 4.90772 5.31106 4.79048 N.3 0 **** 0
76 O1 2.9935 1.37463 6.71103 O.3 0 **** 0	76 O4 2.60414 3.61679 6.47582 O.3 0 **** 0	76 N2 3.48939 4.16391 2.46438 N.3 0 **** 0
77 O2 2.78701 2.5434 9.04867 O.3 0 **** 0	77 O5 3.03391 1.98393 8.63059 O.3 0 **** 0	77 H2A 2.95173 3.81958 1.65385 H 0 **** 0
78 O3 4.3008 4.1226 9.4051 O.3 0 **** 0	78 N1 8.09936 1.55289 6.31246 N.3 0 **** 0	78 H2B 3.84052 3.51088 3.17233 H 0 **** 0
79 O4 5.11791 7.20313 5.8547 O.3 0 **** 0	79 N2 3.41039 2.80762 5.95013 N.3 0 **** 0	79 C1 4.30639 6.08895 3.77042 C.3 0 **** 0
80 O5 5.39831 6.40661 3.80061 O.3 0 **** 0	80 C1 5.34558 1.29628 8.81283 C.3 0 **** 0	80 C2 4.38237 7.49262 3.89235 C.3 0 **** 0
	81 H1 5.20684 0.953628 9.83481 H 0 **** 0	81 H2 4.87833 7.90157 4.76736 H 0 **** 0
	82 C2 6.59366 1.19029 8.22698 C.3 0 **** 0	82 C3 3.86116 8.31756 2.92485 C.3 0 **** 0
	83 H2 7.43188 0.769369 8.77791 H 0 **** 0	83 H3 3.94678 9.39688 3.03792 H 0 **** 0
	84 C3 6.78416 1.63944 6.91595 C.3 0 **** 0	84 C4 3.24746 7.74425 1.78991 C.3 0 **** 0
	85 C4 5.73094 2.16234 6.1734 C.3 0 **** 0	85 H4 2.83556 8.38135 1.00699 H 0 **** 0
	86 H4 5.87222 2.47357 5.14025 H 0 **** 0	86 C5 3.14591 6.37983 1.6542 C.3 0 **** 0
	87 C5 4.48684 2.27398 6.77273 C.3 0 **** 0	87 H5 2.64982 5.94043 0.787773 H 0 **** 0
	88 C6 4.25933 1.85914 8.11523 C.3 0 **** 0	88 C6 3.65176 5.48899 2.64294 C.3 0 **** 0
	89 C7 2.76119 1.3819 9.93002 C.3 0 **** 0	89 O3 2.133 5.64938 5.97038 O.3 0 **** 0
	90 H7A 3.35239 1.87574 10.7143 H 0 **** 0	90 O4 1.17462 7.61685 6.32298 O.3 0 **** 0
	91 H7B 1.69418 1.54053 10.1027 H 0 **** 0	91 O5 0.141236 9.9602 2.17602 O.3 0 **** 0

	92 H7C 2.9852 0.308956 9.89603 H 0 **** 0	92 O6 -1.30884 8.92252 0.848113 O.3 0 **** 0
	93 O6 2.22598 -0.0407567 12.6278 O.3 0 **** 0	93 O7 0.632362 4.12718 4.42036 O.3 0 **** 0
	94 O7 0.333904 1.06585 12.2722 O.3 0 **** 0	94 N3 1.37235 6.58858 5.62482 N.3 0 **** 0
	95 O8 4.33301 0.0930243 16.9242 O.3 0 **** 0	95 N4 -0.467228 8.92708 1.78628 N.3 0 **** 0
	96 O9 3.44477 1.27691 18.5768 O.3 0 **** 0	96 C7 0.711847 6.50169 4.33536 C.3 0 **** 0
	97 N3 1.27332 0.683533 13.0107 N.3 0 **** 0	97 C8 0.428446 7.70007 3.69875 C.3 0 **** 0
	98 N4 3.43391 0.830669 17.4016 N.3 0 **** 0	98 H8 0.676808 8.64264 4.17959 H 0 **** 0
	99 C8 1.25234 1.09992 14.418 C.3 0 **** 0	99 C9 -0.186737 7.67384 2.4552 C.3 0 **** 0
	100 C9 0.128166 1.75543 14.9227 C.3 0 **** 0	100 C10 -0.535775 6.46482 1.84731 C.3 0 **** 0
	101 H9 -0.723363 1.95234 14.2744 H 0 **** 0	101 H10 -0.999294 6.46528 0.863173 H 0 **** 0
	102 C10 0.10692 2.11676 16.2674 C.3 0 **** 0	102 C11 -0.26086 5.27301 2.49048 C.3 0 **** 0
	103 H10 -0.763462 2.62601 16.6764 H 0 **** 0	103 H11 -0.541933 4.3397 2.01337 H 0 **** 0
	104 C11 1.19004 1.82572 17.0932 C.3 0 **** 0	104 C12 0.372362 5.25153 3.74597 C.3 0 **** 0
	105 H11 1.17068 2.08397 18.1499 H 0 **** 0	105 C13 0.237866 2.8649 3.83518 C.3 0 **** 0
	106 C12 2.29626 1.17273 16.5441 C.3 0 **** 0	106 H13A 0.572682 2.10591 4.55067 H 0 **** 0
	107 C13 2.35315 0.79847 15.2071 C.3 0 **** 0	107 H13B -0.855354 2.82408 3.72072 H 0 **** 0
	108 H13 3.21721 0.28731 14.7927 H 0 **** 0	108 H13C 0.737863 2.72589 2.86441 H 0 **** 0
	109 O1 0.0217944 2.83134 1.3373 O.3 0 **** 0	109 O1 5.60472 14.6753 -1.03293 O.3 0 **** 0
	110 O2 0.715045 4.08278 3.03355 O.3 0 **** 0	110 O2 6.20653 12.8196 0.024242 O.3 0 **** 0
	111 O3 5.65253 4.38171 3.50183 O.3 0 **** 0	111 N1 6.18186 14.0916 -0.0548824 N.3 0 **** 0
	112 O4 6.40521 5.55753 1.77307 O.3 0 **** 0	112 N2 7.60199 12.9481 2.27198 N.3 0 **** 0
	113 O5 5.97526 3.92244 -0.380079 O.3 0 **** 0	113 H2A 8.14146 12.6055 3.08203 H 0 **** 0
	114 N1 0.911234 3.49094 1.93885 N.3 0 **** 0	114 H2B 7.25106 12.2935 1.56544 H 0 **** 0
	115 N2 5.60056 4.74749 2.30017 N.3 0 **** 0	115 C1 6.78418 14.8713 0.962539 C.3 0 **** 0
	116 C1 3.66613 3.22683 -0.558768 C.3 0 **** 0	116 C2 6.70731 16.2748 0.83801 C.3 0 **** 0
	117 H1 3.80483 2.88392 -1.58062 H 0 **** 0	117 H2 6.20944 16.6823 -0.0365133 H 0 **** 0

	118 C2 2.41824 3.12212 0.0269607 C.3 0 **** 0	118 C3 7.22901 17.1013 1.80356 C.3 0 **** 0
	119 H2 1.58014 2.6993 -0.522674 H 0 **** 0	119 H3 7.14125 18.1803 1.68929 H 0 **** 0
	120 C3 2.22723 3.57487 1.33663 C.3 0 **** 0	120 C4 7.84509 16.5297 2.93774 C.3 0 **** 0
	121 C4 3.28005 4.09887 2.07908 C.3 0 **** 0	121 H4 8.25861 17.1682 3.71839 H 0 **** 0
	122 H4 3.13863 4.41006 3.1122 H 0 **** 0	122 C5 7.94738 15.1656 3.07548 C.3 0 **** 0
	123 C5 4.52373 4.21184 1.47913 C.3 0 **** 0	123 H5 8.44432 14.728 3.94252 H 0 **** 0
	124 C6 4.75097 3.79463 0.13734 C.3 0 **** 0	124 C6 7.43916 14.2728 2.09052 C.3 0 **** 0
	125 C7 6.24681 3.31882 -1.67898 C.3 0 **** 0	125 O3 1.56871 14.4383 -0.56819 O.3 0 **** 0
	126 H7A 5.6567 3.81368 -2.46344 H 0 **** 0	126 O4 2.52198 16.4084 -0.917204 O.3 0 **** 0
	127 H7B 7.31413 3.47492 -1.85229 H 0 **** 0	127 O5 3.56503 18.7501 3.23009 O.3 0 **** 0
	128 H7C 6.02024 2.24645 -1.6448 H 0 **** 0	128 O6 4.9984 17.7054 4.57078 O.3 0 **** 0
	129 O6 6.78098 1.894 17.5368 O.3 0 **** 0	129 O7 3.06258 12.9139 0.991232 O.3 0 **** 0
	130 O7 8.66863 3.00785 17.8932 O.3 0 **** 0	130 N3 2.32717 15.3785 -0.220465 N.3 0 **** 0
	131 O8 4.67262 2.03437 13.2396 O.3 0 **** 0	131 N4 4.16701 17.7145 3.62331 N.3 0 **** 0
	132 O9 5.56967 3.20581 11.583 O.3 0 **** 0	132 C7 2.98862 15.2909 1.06866 C.3 0 **** 0
	133 N3 7.73263 2.61935 17.1536 N.3 0 **** 0	133 C8 3.27165 16.4894 1.70648 C.3 0 **** 0
	134 N4 5.57618 2.76542 12.7605 N.3 0 **** 0	134 H8 3.02136 17.4322 1.22716 H 0 **** 0
	135 C8 7.75602 3.03099 15.7451 C.3 0 **** 0	135 C9 3.88886 16.4633 2.94923 C.3 0 **** 0
	136 C9 8.88017 3.68778 15.242 C.3 0 **** 0	136 C10 4.23717 15.2542 3.55691 C.3 0 **** 0
	137 H9 9.73066 3.88587 15.8913 H 0 **** 0	137 H10 4.70097 15.2542 4.54079 H 0 **** 0
	138 C10 8.90189 4.05167 13.8981 C.3 0 **** 0	138 C11 3.95943 14.0625 2.91582 C.3 0 **** 0
	139 H10 9.77193 4.5623 13.49 H 0 **** 0	139 H11 4.23796 13.1301 3.39632 H 0 **** 0
	140 C11 7.81936 3.76161 13.0713 C.3 0 **** 0	140 C12 3.32593 14.0405 1.66034 C.3 0 **** 0
	141 H11 7.83888 4.0227 12.0153 H 0 **** 0	141 C13 3.45644 11.6537 1.58272 C.3 0 **** 0
	142 C12 6.71402 3.10588 13.6187 C.3 0 **** 0	142 H13A 3.12348 10.8918 0.869543 H 0 **** 0
	143 C13 6.65679 2.72816 14.9548 C.3 0 **** 0	143 H13B 4.5494 11.6135 1.69956 H 0 **** 0

		144 H13 5.79252 2.21635 15.3678 H 0 **** 0	144 H13C 2.95411 11.5186 2.55263 H 0 **** 0
Bond	1 1 16 1	1 1 6 1	1 1 3 1
	2 1 8 1	2 2 6 1	2 2 3 1
	3 1 2 1	3 3 7 1	3 3 7 1
	4 2 3 1	4 4 7 1	4 4 16 1
	5 2 14 1	5 5 16 1	5 4 5 1
	6 3 5 1	6 5 17 1	6 4 6 1
	7 3 4 1	7 6 12 1	7 7 8 1
	8 5 6 1	8 7 15 1	8 7 16 1
	9 5 15 1	9 8 10 1	9 8 10 1
	10 6 8 1	10 8 16 1	10 8 9 1
	11 6 7 1	11 8 9 1	11 10 12 1
	12 8 9 1	12 10 12 1	12 10 11 1
	13 10 16 1	13 10 11 1	13 12 14 1
	14 10 11 1	14 12 13 1	14 12 13 1
	15 10 12 1	15 13 15 1	15 14 16 1
	16 10 13 1	16 13 14 1	16 14 15 1
	17 14 18 1	17 15 16 1	17 17 22 1
	18 14 17 1	18 17 18 1	18 18 22 1
	19 15 20 1	19 17 19 1	19 19 23 1
	20 15 19 1	20 17 20 1	20 20 23 1
	21 21 36 1	21 21 25 1	21 21 32 1
	22 21 28 1	22 22 25 1	22 21 33 1
	23 21 22 1	23 23 26 1	23 22 24 1
	24 22 23 1	24 24 26 1	24 23 27 1
	25 22 34 1	25 25 27 1	25 24 25 1

26 23 25 1	26 26 34 1	26 24 32 1
27 23 24 1	27 27 35 1	27 25 27 1
28 25 26 1	28 27 28 1	28 25 26 1
29 25 35 1	29 28 30 1	29 27 28 1
30 26 28 1	30 28 29 1	30 28 30 1
31 26 27 1	31 30 32 1	31 28 29 1
32 28 29 1	32 30 31 1	32 30 32 1
33 30 36 1	33 32 34 1	33 30 31 1
34 30 31 1	34 32 33 1	34 33 34 1
35 30 32 1	35 34 35 1	35 33 35 1
36 30 33 1	36 35 36 1	36 33 36 1
37 34 38 1	37 37 42 1	37 37 39 1
38 34 37 1	38 38 42 1	38 38 39 1
39 35 40 1	39 39 43 1	39 39 43 1
40 35 39 1	40 40 43 1	40 40 52 1
41 41 56 1	41 41 52 1	41 40 41 1
42 41 48 1	42 41 53 1	42 40 42 1
43 41 42 1	43 42 48 1	43 43 44 1
44 42 43 1	44 43 51 1	44 43 52 1
45 42 54 1	45 44 46 1	45 44 46 1
46 43 45 1	46 44 52 1	46 44 45 1
47 43 44 1	47 44 45 1	47 46 48 1
48 45 46 1	48 46 48 1	48 46 47 1
49 45 55 1	49 46 47 1	49 48 50 1
50 46 48 1	50 48 49 1	50 48 49 1
51 46 47 1	51 49 51 1	51 50 52 1

52 48 49 1	52 49 50 1	52 50 51 1
53 50 56 1	53 51 52 1	53 53 58 1
54 50 51 1	54 53 54 1	54 54 58 1
55 50 52 1	55 53 55 1	55 55 59 1
56 50 53 1	56 53 56 1	56 56 59 1
57 54 58 1	57 57 61 1	57 57 68 1
58 54 57 1	58 58 61 1	58 57 69 1
59 55 60 1	59 59 62 1	59 58 60 1
60 55 59 1	60 60 62 1	60 59 63 1
61 61 76 1	61 61 63 1	61 60 61 1
62 61 68 1	62 62 70 1	62 60 68 1
63 61 62 1	63 63 71 1	63 61 63 1
64 62 63 1	64 63 64 1	64 61 62 1
65 62 74 1	65 64 66 1	65 63 64 1
66 63 65 1	66 64 65 1	66 64 66 1
67 63 64 1	67 66 68 1	67 64 65 1
68 65 66 1	68 66 67 1	68 66 68 1
69 65 75 1	69 68 70 1	69 66 67 1
70 66 68 1	70 68 69 1	70 69 70 1
71 66 67 1	71 70 71 1	71 69 71 1
72 68 69 1	72 71 72 1	72 69 72 1
73 70 76 1	73 73 78 1	73 73 75 1
74 70 71 1	74 74 78 1	74 74 75 1
75 70 72 1	75 75 79 1	75 75 79 1
76 70 73 1	76 76 79 1	76 76 88 1
77 74 78 1	77 77 88 1	77 76 77 1

78 74 77 1	78 77 89 1	78 76 78 1
79 75 80 1	79 78 84 1	79 79 80 1
80 75 79 1	80 79 87 1	80 79 88 1
	81 80 82 1	81 80 82 1
	82 80 88 1	82 80 81 1
	83 80 81 1	83 82 84 1
	84 82 84 1	84 82 83 1
	85 82 83 1	85 84 86 1
	86 84 85 1	86 84 85 1
	87 85 87 1	87 86 88 1
	88 85 86 1	88 86 87 1
	89 87 88 1	89 89 94 1
	90 89 90 1	90 90 94 1
	91 89 91 1	91 91 95 1
	92 89 92 1	92 92 95 1
	93 93 97 1	93 93 104 1
	94 94 97 1	94 93 105 1
	95 95 98 1	95 94 96 1
	96 96 98 1	96 95 99 1
	97 97 99 1	97 96 97 1
	98 98 106 1	98 96 104 1
	99 99 107 1	99 97 99 1
	100 99 100 1	100 97 98 1
	101 100 102 1	101 99 100 1
	102 100 101 1	102 100 102 1
	103 102 104 1	103 100 101 1

104 102 103 1	104 102 104 1
105 104 106 1	105 102 103 1
106 104 105 1	106 105 106 1
107 106 107 1	107 105 107 1
108 107 108 1	108 105 108 1
109 109 114 1	109 109 111 1
110 110 114 1	110 110 111 1
111 111 115 1	111 111 115 1
112 112 115 1	112 112 124 1
113 113 124 1	113 112 113 1
114 113 125 1	114 112 114 1
115 114 120 1	115 115 116 1
116 115 123 1	116 115 124 1
117 116 118 1	117 116 118 1
118 116 124 1	118 116 117 1
119 116 117 1	119 118 120 1
120 118 120 1	120 118 119 1
121 118 119 1	121 120 122 1
122 120 121 1	122 120 121 1
123 121 123 1	123 122 124 1
124 121 122 1	124 122 123 1
125 123 124 1	125 125 130 1
126 125 126 1	126 126 130 1
127 125 127 1	127 127 131 1
128 125 128 1	128 128 131 1
129 129 133 1	129 129 140 1

		130 130 133 1	130 129 141 1
		131 131 134 1	131 130 132 1
		132 132 134 1	132 131 135 1
		133 133 135 1	133 132 133 1
		134 134 142 1	134 132 140 1
		135 135 143 1	135 133 135 1
		136 135 136 1	136 133 134 1
		137 136 138 1	137 135 136 1
		138 136 137 1	138 136 138 1
		139 138 140 1	139 136 137 1
		140 138 139 1	140 138 140 1
		141 140 142 1	141 138 139 1
		142 140 141 1	142 141 142 1
		143 142 143 1	143 141 143 1
		144 143 144 1	144 141 144 1
Crysin	3.868 13.533 15.321 90 95.302 90 1 1	18.862 3.7905 21.8197 90 106.703 90 1 1	7.583 17.552 11.492 90 95.214 90 1 1

Table S3 Lattice parameters comparison between optimized structures under COMPASS force field and experimental data

		DNAN	DNAN/DNB	DNAN/NA
a (Å)	COMPASS	3.73	18.81	7.43
	Exp.	3.91	18.81	7.43
	Error (%)	4.68	-0.02	0.04
b (Å)	COMPASS	14.00	3.87	17.57
	Exp.	13.78	3.87	17.57
	Error (%)	-1.60	-0.01	-0.01
c (Å)	COMPASS	15.20	21.91	11.49

	Exp.	15.42	21.91	11.49
	Error (%)	1.45	0.00	-0.02
α (°)	COMPASS	90.04	90.00	90.00
	Exp.	90.00	90.00	90.00
	Error (%)	-0.05	0.00	0.00
β (°)	COMPASS	99.32	106.70	95.21
	Exp.	95.31	106.70	95.21
	Error (%)	-4.21	0.00	0.00
γ (°)	COMPASS	90.01	90.00	90.00
	Exp.	90.00	90.00	90.00
	Error (%)	-0.01	0.00	0.00

Code S1 Codes for calculating the electronic structures

```
my $results = Modules->CASTEP->Energy->Run($system, Settings(
        UseCustomEnergyCutoff => "Yes",
        EnergyCutoff => 600,
        SCFConvergence => 2e-005,
        MaximumSCFCycles => 500,
        UseDFTD => "Yes",
        DFTDMethod => "Grimme",
        UseInsulatorDerivation => "Yes",
        Pseudopotentials => "Norm-conserving",
```

```
        CalculateDensityDifference => "FieldAndIsosurface",
        CalculateBondOrder => "Mulliken",
        CalculateCharge => "Mulliken", # or "Hirshfeld"
));
```

Code S2 Codes for calculating T_m

```
#!perl Rate=5 K/step, timestep=1 fs/step

use strict;
use Getopt::Long;
use MaterialsScript qw(:all);

my $NVT_Structure;
my $finalStructure;
my $doc_1;
my $doc_2;
my $temperature;


my $j=0;
for (my $n=0; $n<=30; ++$n)
{
my $temperature=380+$n*5;

$doc_1=$Documents{"$j.xsd"};
```

```
my $results = Modules->Forcite->Dynamics->Run($doc_1, Settings(
        Quality => 'Fine',
        CurrentForcefield => "COMPASS",
        AssignForcefieldTypes => "No",
        ChargeAssignment => "Use current",
        Ensemble3D => "NVT",
        Temperature => $temperature,
        Pressure => 0.0001,
        NumberOfSteps => 50000,
        TimeStep => 1,
        TrajectoryFrequency => 100
        ));

my $outTrajectory_1 = $results->Trajectory;
$NVT_Structure=$results->Structure;
$j=$j+1;
$NVT_Structure->Saveas("$j.xsd");


$doc_2=$Documents{"$j.xsd"};

my $results = Modules->Forcite->Dynamics->Run($doc_2, Settings(
        Quality => 'Fine',
        CurrentForcefield => "COMPASS",
        AssignForcefieldTypes => "No",
        ChargeAssignment => "Use current",
```

```
        Ensemble3D => "NPT",
        Temperature => $temperature,
        Pressure => 0.0001,
        NumberOfSteps => 150000,
        TimeStep => 1,
        TrajectoryFrequency => 100
        ));


my $outTrajectory_2 = $results->Trajectory;
$finalStructure=$results->Structure;
$j=$j+1;
$finalStructure->Saveas("$j.xsd");

}
```
